# Efficacy and safety of Jian-Pi Huo-Xue granule for non-alcoholic fatty liver disease: study protocol for a randomized, double-blind, placebo-controlled trial

**DOI:** 10.1186/s13063-022-06393-8

**Published:** 2022-06-02

**Authors:** Yuanlong Sun, Gaofeng Chen, Si Chen, Yanjie Wang, Yiyang Hu, Yu Zhao

**Affiliations:** 1 Key Laboratory of Liver and Kidney Diseases (Ministry of Education), Institute of Liver Diseases, Shanghai Key Laboratory of Traditional Chinese Clinical Medicine, Shuguang Hospital Affiliated to Shanghai University of Traditional Chinese Medicine, No. 528 Zhangheng Road, Pudong New Area, Shanghai, 201203 China; 2 Institute of Clinical Pharmacology, Shanghai University of Traditional Chinese Medicine, Ministry of Education, Shanghai, 201203 China

**Keywords:** Jian-Pi Huo-Xue granule, Non-alcoholic fatty liver disease, Traditional Chinese medicine, Randomized controlled trial

## Abstract

**Background:**

Non-alcoholic fatty liver disease (NAFLD) has become the most prevalent form of chronic liver disease, with a global prevalence of 25% worldwide, but a consensus treatment is still lacking. Previous studies have shown that Jian-Pi Huo-Xue granules (JPHX) can reduce hepatic steatosis in ultrasound images, but lacked quantitative observation in imagined liver fat content. This study aimed to refine the efficacy and safety assessment of JPHX for NAFLD with magnetic resonance imaging-proton density fat fraction (MRI-PDFF) as the primary outcome.

**Methods:**

This is a randomized, double-blind, placebo-controlled clinical trial. The trial will enrol 84 NAFLD participants who will be equally randomized to receive either JPHX or a placebo for 24 weeks. Follow-up will be performed 12 weeks after the intervention. The primary outcome will be the change from baseline to week 24 in MRI-PDFF. Secondary outcomes will be the body weight, body mass index (BMI), waist circumference, waist-to-hip ratio (WHR), serum liver function, blood lipids and glucose-related indicators, quality of life measurement health survey, and traditional Chinese medicine (TCM) syndrome scale. Outcomes will be monitored at baseline, 12 weeks and 24 weeks after enrolment. Adverse events occurring in this trial will be managed and recorded promptly.

**Discussion:**

We designed a clinical trial for the treatment of NAFLD using JPHX, a TCM formulation that has been shown to have a positive effect on hepatic steatosis in a previous self-controlled trial. This trial will use a more recognized and quantitative imaging approach to demonstrate the efficacy of JPHX in the treatment of NAFLD and observe its safety to provide clinical evidence for its translational applications.

**Trial registration:**

Chinese Clinical Trial Registry ChiCTR2100046132. Registered on 4 May 2021.

## Administrative information

Note: the numbers in curly brackets in this protocol refer to SPIRIT checklist item numbers. The order of the items has been modified to group similar items (see http://www.equator-network.org/reporting-guidelines/spirit-2013-statement-defining-standard-protocol-items-for-clinical-trials/).

**Table:** 

Title {1}	Efficacy and safety of Jian-Pi Huo-Xue granule for Non-alcoholic Fatty Liver Disease: study protocol for a randomized, double-blind, placebo-controlled trial
Trial registration {2a and 2b}.	Chinese Clinical Trial Registry, ID: ChiCTR2100046132. Registered on May 4, 2021.
Protocol version {3}	December 31, 2020, version 1.0
Funding {4}	This study was funded by the Science and Technology Commission of Shanghai Municipality (20Y21901700).
Author details {5a}	(1) Yuanlong Sun, MD, Ph.D., E-mail: sunyuanlong@shutcm.edu.cn; (2) Gaofeng Chen, RA, E-mail: E-mail: gaofengchen06@126.com; (3) Si Chen, MPH, E-mail: 240076589@qq.com; (4) Yanjie Wang, E-mail: 1440327036@qq.com; (5) Yiyang Hu, MD, Ph.D., E-mail: yyhuliver@163.com; (6) Yu Zhao, MD, Ph.D., E-mail: cathy150@139.com;
Name and contact information for the trial sponsor {5b}	Sponsor: Shuguang Hospital of Shanghai University of Traditional Chinese Medicine, Zhangheng Road 528, Pudong New Area, Shanghai, China Coordinating Investigator (contact): Prof. Dr. Yu Zhao Institute of Liver Disease, Shuguang Hospital of Shanghai University of Traditional Chinese Medicine Zhangheng Road 528 Pudong New Area, Shanghai, China Email: cathy150@139.com;
Role of sponsor {5c}	The sponsor has no role in the design of the study, in the collection, analysis, and interpretation of the data, and the writing of the manuscript.

## Introduction

Non-alcoholic fatty liver disease (NAFLD) has become the most common form of chronic liver disorder worldwide [1, 2]. NAFLD can be histologically categorized into the nonalcoholic fatty liver (NAFL) or nonalcoholic steatohepatitis (NASH) [3]. NAFL is histologically characterized by hepatic steatosis over 5% without evidence of hepatocellular injury. Histologically, NASH is characterized by hepatic steatosis over 5% with hepatocyte ballooning degeneration and inflammation, with or without fibrosis [4]. The prevalence of NAFLD is increasing year by year, an estimated 25% of the global population has NAFLD [5, 6]. The incidence of NASH is projected to increase by up to 56% in 2030 [7], and NASH is predicted to be the main cause of hepatocellular carcinoma in liver transplants [2, 8]. China experienced an unexpected rapid increase in the burden of NAFLD over a short period [9]. According to the epidemiological survey in China, the prevalence of NAFLD in the overall population is 29.6% [10]. Patients with NAFLD have occult onset and chronic progression of liver disorders that are usually associated with metabolic syndrome, including obesity, abnormal lipid metabolism, hypertension diabetes, and insulin resistance [11–13]. An international expert consensus has reached the concept of metabolic associated fatty liver disease (MAFLD) to reflect the hepatic manifestations of multisystem disorder [14–16].

The prevalence of NAFLD is associated with sedentary behaviour, low physical activity, and poor diet lifestyle, lifestyle modifications such as diet and exercise are recommended as the primary intervention for NAFLD [2]. However, lifestyle interventions can be useful but hard for effective long-term maintenance. Current therapeutic landscape for NAFLD mainly focuses on interventions in energy intake, energy processing, lipotoxic liver injury, and inflammation and fibrosis leading to cirrhosis. However, all drugs are still in preclinical development or clinical trials to evaluate efficacy [17], and there are currently no FDA-approved drugs for NAFLD.

The prevention and treatment of chronic liver disease by traditional Chinese medicine (TCM) have a long history and extensive clinical application in China. Jian-Pi Huo-Xue granules (JPHX) consists of 8 herbs and is a TCM experience formula for the treatment of alcoholic liver disease (ALD) and NAFLD [18, 19]. Our previous experiments showed that JPHX attenuates intestinal mucosal epithelial cell injury, decreases intestinal endotoxin leakage [20–22], and ameliorates hepatic steatosis and inflammation [23, 24] in ALD rats induced by a Lieber-DeCarli liquid diet. JPHX also has a hepatoprotective effect in NAFLD rats fed a methionine-choline-deficient diet [25]. “Leaky gut” is a common pathological feature of ALD and NALFD; the intestinal barrier protection of JPHX in ALD rats also suggests a potential therapeutic effect on NAFLD.

In a previous self-controlled pilot study, He et al. treated NAFLD patients with JPHX and found that JPHX significantly improved imaged liver fat assessed by ultrasound and reduced liver dysfunction [26, 27]. However, the pilot study lacks quantitative observations of the primary efficacy outcome. Therefore, we designed a randomized, double-blind, placebo-controlled trial to investigate the clinical efficacy and safety of JPHX as a treatment for NAFLD using magnetic resonance imaging-based proton density fat fraction (MRI-PDFF) as the primary outcome.

## Methods

### Study design

**Fig. 1:**
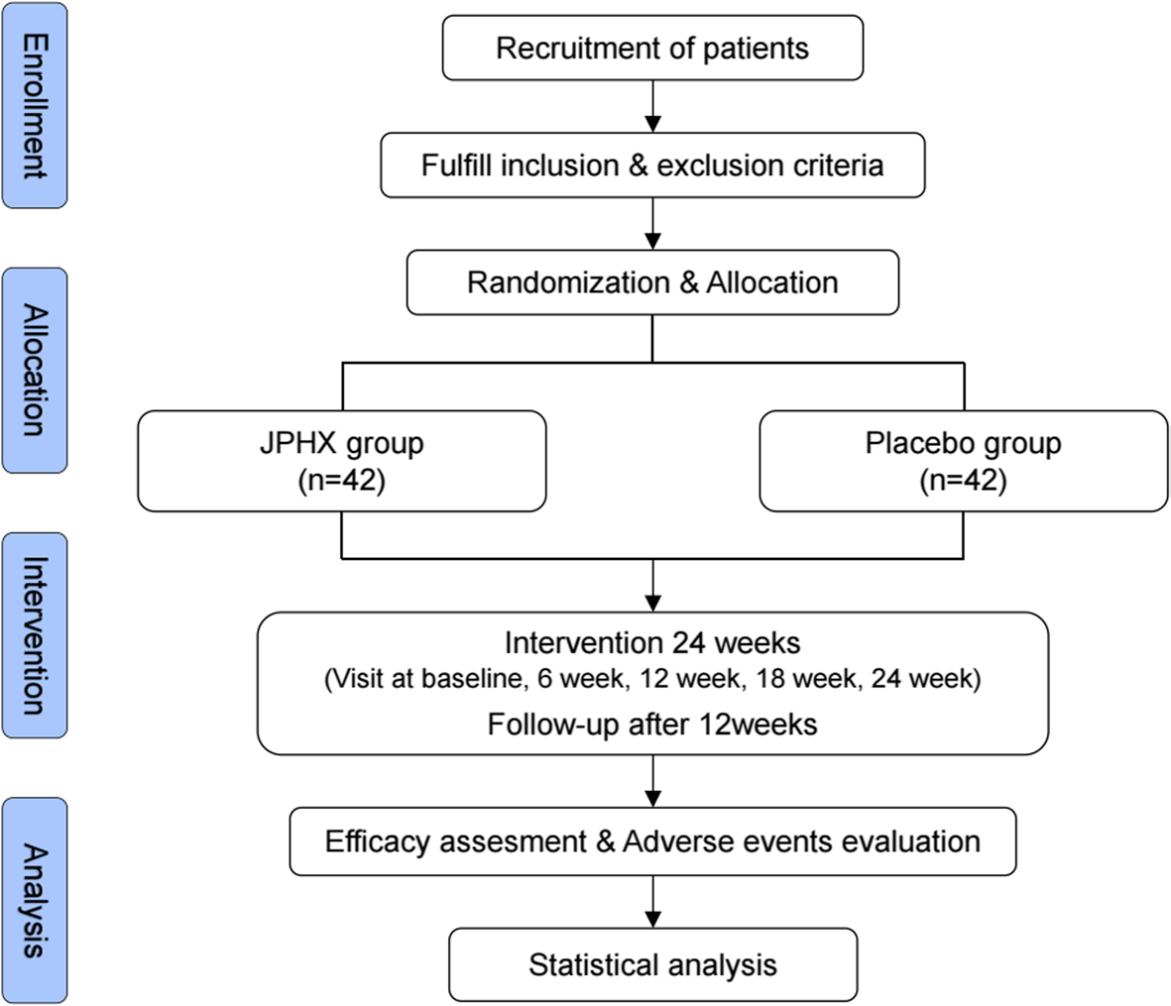
Flow chart of this trial

This is a randomized, double-blind, placebo-controlled clinical trial. The flow chart of the trial is shown in Fig. 1. This trial is conducted in Shuguang Hospital Affiliated with Shanghai University of Traditional Chinese Medicine.

### Interventions

Participants who met the inclusion criteria and signed an informed consent form will be randomly assigned to the JPXH group or the placebo-controlled group.

JPHX group: JPHX granules (6.39 g/pack × 2 packs, bid)

Placebo group: JPHX-placebo granules (2 packs, bid)

JPHX granules and placebo are recommended to be diluted in water and taken half an hour after meals. JPHX granules are composed of 8 kinds of Chinese herbs, including *Atractylodes macrocephala Koidz.* (Bai Zhu), *Salvia miltiorrhiza Bge.* (Dan Shen), *Citrus aurantium L.* (Zhi Qiao), *Paeonia lactiflora Pall.* (Bai Shao), *Pueraria lobata (Willd.) Ohwi* (Ge Gen), *Alisma orientalis (Sam.) Juzep.* (Ze Xie), *Schisandrae Chinensis Fructus.* (Wu Wei Zi), and *Curcuma Longa L.* (Jiang Huang) [19]. The placebo granules will be masked with the same packaging and similar taste compared to JPHX. Both JPHX and placebo are manufactured and packaged by Jiangyin Tianjiang Pharm Co., Ltd, Jiangyin city, Jiangsu, China.

Participants will receive regular health education, including diet control, exercise, and behaviour modification. ① Diet adjustment: diets with low-sugar, low-fat, and high-vitamin are recommended. ② Strengthen physical exercise: moderate-equivalent-intensity exercise, more than 30 min each time, 4 times a week. ③ Adjust mood and maintain a good mood; ④ During the trial, other TCM herbs and Western medicines mainly used to lower lipids or treat liver damage are prohibited.

### Withdrawal, dropout, and discontinuation

Participants will be advised to discontinue participation in the event of a serious adverse event (SAE), or a condition during the course that does not meet the requirements of this trial, such as pregnancy or taking illicit drugs. There are no special criteria for modifying allocated interventions. Participants are free to withdraw at any time and the reason for withdrawal will be investigated. The reason and its connection with this trial will be recorded after investigation. If a moderate or severe adverse reaction determined to be related to the clinical trial occurs in more than 25% of the total participants, or if the required number of participants is not fully enrolled within the schedule, this clinical trial may be terminated.

### Strategies to improve adherence to interventions

Investigators will conduct regular phone calls or connect participants via communication software to ensure compliance. We will evaluate the compliance to the treatment by granules packet count, patients who complete 80% of the recommended treatment will be considered in compliance.

### Post-trial care

Patients that are enrolled into the trial are covered by indemnity for negligent harm in accordance with national laws and regulations. If the participant has the treatment and examination required for other diseases at the same time, it will not be included in the free scope.

### Outcomes

The primary outcome is the relative change of MRI-PDFF from baseline to 24 weeks.

The secondary outcomes include changes in ① body weight, body mass index (BMI), waist-to-hip ratio (WHR); ② serum liver function including total bilirubin (TBIL), direct bilirubin (DBIL), indirect bilirubin (IDBIL), ALT, aspartate aminotransferase (AST) [16], gamma-glutamyl transpeptidase (GGT), alkaline phosphatase (ALP), albumin (ALB) [28], pre-albumin (Pre-ALB), and total bile acid (TBA); ③ fasting serum lipids; ④ blood glucose-related indicators, such as fasting plasma glucose (FPG), fasting insulin (FINS), and Homeostasis model assessment – insulin resistance (HOMA-IR). All mentioned serum biochemical markers will be measured at baseline, 12 weeks and 24 weeks after randomization. ⑤ Health related quality of life (SF-36). ⑥ Chronic Liver Disease Questionnaire (CLDQ), ⑦ TCM Syndrome Score Scale (TCMSSS).

Blood routine, urine routine, blood urea nitrogen, creatinine, uric acid, and SAEs are included as safety outcomes in this trial.

### Sample size justification

A previous pilot trial in Shuguang Hospital used comprehensive scores of abdominal ultrasounds to evaluate efficacy. The score includes the evaluation of 7 indicators such as liver shape, contour, echo, and vascular structure. Each indicator is divided into 4 grades: normal, mild, moderate, and severely abnormal. Twenty-six patients (65%) experienced a 1-grade or more decrease in at least 3 indicators after treatment with JPHX compared to baseline [27, 29]. In this trial, a 26% relative reduction from baseline in liver MRI-PDFF will be expected in 60% of JPHX-treated cases compared to 30% in the placebo control group. Assuming *α* = 0.05, *β* = 0.2, *π*_*T*_ = 0.6, *π*_*C*_ = 0.3, *δ* = 0.01, *n*_*T*_ : *n*_*C*_ = 1:1. According to the optimal sample size calculation formula, each group will get 33 samples. Considering the dropout rate of 20%, a minimum of 42 individuals will be obtained per group.

$${\mathrm{n}}_{\mathrm{T}}={\mathrm{n}}_{\mathrm{C}}=\frac{{\left({z}_{1-\alpha }+{z}_{1-\beta}\right)}^2\left[{\pi}_T\left(1-{\pi}_T\right)+{\pi}_C\left(1-{\pi}_C\right)\right]}{{\left[\left({\pi}_T-{\pi}_C\right)-\delta \right]}^2}$$

## Study procedures

### Participants

#### Diagnostic criteria

The diagnosis of NAFLD is according to the “Guidelines of prevention and treatment for nonalcoholic fatty liver disease (2018, China)” promulgated by the National Workshop on Fatty Liver and Alcoholic Liver Disease, Chinese Society of Hepatology, Chinese Medical Association [30]. In this trial, NAFLD participants are characterized by histopathology-proven significant hepatic steatosis or imaging-proven fatty liver by MRI-PDFF (MRI-PDFF>5%), and no history of alcohol or overconsumption (<210 g per week in men and <140 g per week in women during the past 12 months). Liver biopsy is difficult in the NAFLD clinic, recommend to participants to attempt biopsy to determine NASH status based on a voluntary principle or a medical indication.

The diagnosis of TCM syndromes is based on the “Diagnostics of Traditional Chinese Medicine” [31] and the “Consensus opinion on diagnosis and treatment of nonalcoholic fatty liver disease with integrated traditional Chinese and Western Medicine” [32].

#### Inclusion criteria

We will include eligible participants: ① diagnosed with NAFLD; ② aged 18 to 65 years; ③ with imaging evidence of liver MRI-PDFF ≥ 5%; ④ BMI < 35 kg/m^2^; ⑤ willing to follow the scheduled visit plan; ⑥ with serum alanine aminotransferase (ALT) < 5 times the upper normal limit (ULN); and ⑦ and signed informed consent.

#### Exclusion criteria

We will exclude those who: ① regularly took other hepatoprotective drugs within 1 month before screening; ② have taken drugs that may affect lipid metabolism within 3 months before screening, such as tamoxifen, amiodarone, sodium valproate, methotrexate, and glucocorticoid; ③ combined with special conditions that may lead to fatty liver, such as total parenteral nutrition, inflammatory bowel disease, celiac disease, hypothyroidism, Cushing’s syndrome, β-lipoprotein deficiency, lipoatrophic diabetes, and Mauriac syndrome; ④ are diagnosed of ALD (male alcohol intake > 30g/day, female alcohol intake > 20g/day), liver cirrhosis, liver decompensation, and other chronic liver diseases, such as hepatitis B, hepatitis C, autoimmune liver diseases; ⑤ have received liver transplantation; ⑥ combined with unstable controlled type 2 diabetes (HbA1c≥9.5% before enrolment); ⑦ combined with a high or extremely high risk of arteriosclerotic cardiovascular disease (ASCVD); ⑧ have had bariatric surgery in the past year or have taken weight-loss drugs in the past three months, and have lost more than 10% of their body weight; ⑨ have a history of drug or narcotic abuse; or ⑩ are pregnant and lactating or have major primary diseases or malignant tumours.

### Recruitment

Eligible participants will get basic information about this trial through outpatient physicians or the advertisements located in the hospital and communicate with investigators through contact details. Trained researchers will conduct investigations, obtain informed consent, and attending physicians will be responsible for the recruitment. Participants will be informed of the detailed procedures of the trial and shown the informed consent form after being verbally explained the possible benefits and potential risks to ensure that participation is entirely voluntary. Participants will have the option to proceed after signing the informed consent form. During enrolment, disease-related biological markers and imaging examinations will be performed, and the results will be confidentially recorded in the case report form (CRF).

### Study visit overview

**Fig. 2:**
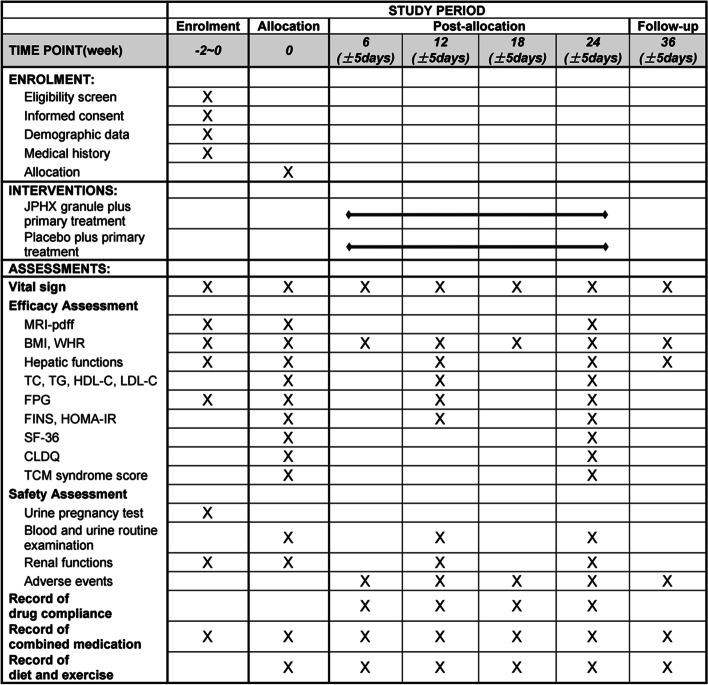
Study schedule of enrolment, interventions, and assessments. Abbreviations: JPHX, Jian-Pi Huo-Xue granule; MRI-PDFF, magnetic resonance imaging - proton density fat fraction; BMI, body mass index; WHR, waist-to-hip ratio; TC, total cholesterol; TG, triglyceride; HDL-C, high-density lipoprotein cholesterol; LDL-C, low-density lipoprotein cholesterol; FPG, fasting plasma glucose; FINS, fasting insulin; HOMA-IR, homeostasis model assessment - insulin resistance; SF-36, Medical Outcomes Study item short-form health survey; CLDQ, Chronic Liver Disease Questionnaire; NAFLD, non-alcoholic fatty liver disease; TCM, traditional Chinese medicine

Five visits including one follow-up are designed in this trial: at 6, 12, 18, 24, and 36 weeks after enrolment. Each time point has 5 days of time flexibility. Participants will have their vital signs checked at each visit, receive health education and medication for the next 6 weeks, and return any remaining drugs from the previous visit. Other concomitant medications and their doses during this period will be recorded in the CRF. Compliance with the treatment will be evaluated by pack count at each visit and serious adverse events will be recorded. MRI-PDFF examinations will be performed at weeks 0 and 24, and laboratory indicators will be measured at weeks 0, 12, and 24. SF-36, CLDQ, and TCMSSS will be filled out at 0 and 24 weeks. The TCMSSS consists of 55 questions about conventional symptoms of TCM syndromes, and each symptom is divided into four grades according to its frequency or degree. These questions are derived from a Patient-Reported Outcome (PRO) questionnaire for patients with hepatitis B virus-related cirrhosis (PHBC-PRO) [33, 34] and are expanded to studies of TCM for the treatment of chronic liver diseases. TCM syndrome diagnosis and its related degree can be reflected according to the combination and degree of positive symptoms. Any questions raised by participants will be answered to facilitate completion. The study schedule is shown in Fig. 2.

### Randomization and concealment

#### Allocation

Enrolled participants will be randomized 1:1 to either the JPHX group or the placebo control group according to a randomization sequence designed by an external statistician using SPSS software (Version 24.0). The sequence will be sealed in opaque envelopes and kept independently as confidential documents.

#### Blinding

All participants and investigators will be blinded to treatment assignments until the study is complete. Personnel unrelated to this trial will complete the preparation of drug blinding and emergency letters. The unblinding procedure will be performed twice. The first unblinding process will break down the assigned groups (Group A and Group B) without a specific intervention name. The statistician will perform statistical analysis without knowledge of the intervention. After analysis, a second unblinding will be conducted to reveal which group (Group A/Group B) is the treatment group/placebo group. All unblinding processes will be recorded. In case of emergency, we will record the reason, time, and place of breaking blindness and will treat it as a withdrawal case.

### Ethical consideration

The clinical trial was approved by the Ethics Committee of Shuguang Hospital (Ethics approval number: 2021-934-09-01) on January 27, 2021. If the protocol is modified during the trial, it needs to be re-approved by the ethics committee before implementation. The process of this trial will follow the ethical regulations of the Declaration of Helsinki and will be monitored by the Institutional Review Board (IRB) of Shuguang Hospital affiliated with Shanghai University of TCM and the Science and Technology Commission of Shanghai Municipality.

### Data management

All collected data will be managed by an Electronic Data Capture (EDC) system program compiled by the Information Technology Department in Shuguang Hospital. Data will be entered and proofread independently by two data administrators. Locked data files will not be changed. Problems found after data locking will be corrected in the statistical analysis state after confirmation. Participant data will be anonymized and all data collected will be kept confidential in this trial.

### Data monitoring and Steering committee

The principal investigators will supervise correct development of the trial. The data monitoring committee of the Clinical Research Center of Shuguang Hospital and the Science and Technology Commission of Shanghai Municipality oversee the trial. The results of this trial will be submitted for publication in peer-reviewed journals and can be disseminated through conference presentations.

### Statistical analysis

Statistical analysis will be performed by an independent researcher. The primary outcome efficacy evaluation will be conducted in the full analysis set [35] according to the intention-to-treatment (ITT) principle. Cases that met the trial protocol had good compliance (> 80%), did not take illicit drugs during the trial, and completed the contents of the CRF regulations will be defined as the Per-Protocol Set. The missing data will be replaced using the last observation carried forward (LOCF) method.

Continuous data will be described using mean ± standard deviation or median (range) depending on the distribution of the data. Comparison of mean or median values of continuous variables from the two groups will be undertaken using an independent *t* test or Wilcoxon rank-sum test. Paired *t* test or Wilcoxon signed-rank test will be used to compare the distribution of pre- and post-treatment changes within each group. Categorical data will be described by frequency counts and constituent ratio and will be compared using the chi-square test/Fisher exact test. All statistical analyses will be conducted with the bilateral test, and *P* value less than or equal to 0.05 will be considered statistically significant. Subgroup analyses will be performed based on information collected to explore the source of potential high heterogeneity.

## Discussion

According to previous studies, JPHX has liver protection in NAFLD rodents. The study by Feng et al. [25] showed that the serum ALT, AST, TC, and TG levels were significantly decreased in JPHX-intervention rats compared with NAFLD rats fed an MCD diet, as was the NAFLD activity score (NAS). JPHX has a direct hepatoprotective effect and reduces the gene and protein expression of tumour necrosis factor-α (TNF-α) in RAW264.7 cells induced by lipopolysaccharide [19]. Therefore, we initiated this randomized, double-blind, placebo-controlled clinical trial to further evaluate the efficacy of JPHX in the treatment of patients with NAFLD.

In addition to liver biopsy, imaging examination is still the main diagnostic method for NAFLD. Abdominal ultrasound is commonly used to screen fatty liver in clinical, but its accuracy is unsatisfactory. The controlled attenuation parameter (CAP) for the non-invasive assessment of hepatic steatosis based on the vibration-controlled transient elastography (VCTE) platform is new and quantitative [36]. CAP can detect hepatic steatosis over 5% and accurately distinguish mild hepatic steatosis from moderate to severe hepatic steatosis. However, the diagnostic accuracy of CAP for fatty liver decreased when BMI > 30 kg/m^2^ and the interquartile range (IQR) > 40 dB/m [36–39].

Considering that it is difficult to perform liver biopsies in fatty liver clinical practice, this trial used the relative change in MRI-PDFF from baseline as the primary outcome. MRI-PDFF is a new MRI technique with more accurate performance than CAP in the non-invasive diagnosis of hepatic steatosis in NAFLD patients [40]. There is a significant correlation between MRI-PDFF values in NAFLD patients and steatosis grades determined by invasive histologic examination [41]. The cut-off criteria for MRI-PDFF to correspond to NAFLD pathological grading are unclear, and MRI-PDFF ≥ 5% or hepatic steatosis ≥ 5% on liver biopsy are commonly used as diagnostic criteria for fatty liver disease [42]. Loomba's study [3] found an absolute decrease of 4.8% or a relative decrease of 26% in MRI-PDFF from baseline was associated with a 1-grade improvement in hepatic steatosis on liver biopsy. Based on this, we used MRI-PDFF <5% at week 24 post-enrolment as the recovery criterion and a 4.8% absolute or 26% relative decrease in MRI-PDFF from baseline as improvement in hepatic steatosis. The liver function, blood lipids and glucose, BMI, waist-hip circumference, and SF-36 score will also be observed as the effects of JPHX on NAFLD patients.

In conclusion, this is a randomized, double-blind, placebo-controlled clinical trial to evaluate the efficacy and safety of JPHX for NAFLD patients. The findings of this trial may provide evidence for a rigorous, large-scale, confirmatory RCT to confirm the efficacy and safety of JPHX for the administration of patients with NAFLD.

## Trial status

The final protocol version is 1.0 and is dated December 31, 2020. This trial has currently recruited the first participant on 20 June 2021.

## Data Availability

The datasets used and/or analysed during the current study are available from the corresponding author on reasonable request.

## Abbreviations

ALD: Alcoholic liver disease
ALP: Alkaline phosphatase
ALT: Aspartate aminotransferase
AST: Gamma-glutamyl transpeptidase
BMI: Body mass index
CRF: Case report form
DRQ: Concentrator data ready queue
FINS: Fasting insulin
FINS: Fasting insulin
FPG: Fasting plasma glucose
GGT: Total bilirubin
HDL-C: High-density lipoprotein cholesterol
HOMA-IR: Homeostasis model assessment - insulin resistance
IDBIL: Indirect bilirubin
JPHX: Jian-Pi Huo-Xue granule
LDL-C: Low-density lipoprotein cholesterol
MRI-PDFF: Magnetic resonance imaging - proton density fat fraction
NAFLD: Non-alcoholic fatty liver disease
NASH: Non-alcoholic steatohepatitis
SF-36: Medical Outcomes Study item short-form health survey
TBA: Total bile acid
TBIL: Direct bilirubinDBIL
TC: Total cholesterol
TCM: Traditional Chinese medicine
ALT: Alanine aminotransferase
TG: Triglyceride
WHR: Waist-to-hip ratio
